# Multiple Sclerosis Masquerading as Post Septorhinoplasty Complication: A Case Report

**DOI:** 10.7759/cureus.19591

**Published:** 2021-11-15

**Authors:** Dana Alolayet, Fahad Alobaid, Muhammad E Ahmed, Khurram Waheed

**Affiliations:** 1 Otolaryngology - Head and Neck Surgery, King Abdulaziz Medical City, Riyadh, SAU; 2 Medical Imaging, King Abdulaziz Medical City, Riyadh, SAU

**Keywords:** facial plastic surgery, nasal trauma, nasal obstruction, multiple sclerosis, septorhinoplasty

## Abstract

This is a case report of a young woman, who after a successful septorhinoplasty procedure, sustained repeated nasal trauma with a subsequent diagnosis of multiple sclerosis (MS) at a large tertiary hospital in Riyadh, Saudi Arabia. A 24-year-old woman with a history of childhood trauma presented with difficulty in breathing and dissatisfaction with her nasal appearance. After a successful and uneventful septorhinoplasty, she required numerous hospital admissions due to multiple episodes of blunt nasal trauma, culminating in clear nasal discharge and neurological symptoms, including dizziness, right-sided paresthesia and difficulty walking. Cerebrospinal fluid (CSF) leak was ruled out by CT brain; however, magnetic resonance imaging (MRI) of the brain and spinal cord showed demyelinating areas in the brain and cervical region of the spinal cord. CSF examination revealed the presence of oligoclonal bands. A neurologist confirmed the diagnosis of MS and initiated treatment, which was well tolerated. The patient is in remission with mild paresthesia in the right hand. Despite the repeated nasal trauma, the septorhinoplasty procedure had an excellent outcome. In conclusion, repeated nasal trauma, especially in the early postoperative period, in addition to procedure failure, may also point to the presence of an uncommon underlying neurological disorder, hitherto undiagnosed. It is therefore important to have an open mind when it comes to the differential diagnosis in such unusual scenarios. In addition, while investigating recurrent nasal trauma, it is extremely important to keep in mind rare neurological conditions, especially in younger patients.

## Introduction

Septorhinoplasty is one of the commonest otolaryngological procedures performed to alleviate nasal obstruction and correct cosmetic defects. Like any surgical procedure, septorhinoplasty itself may be associated with complications including bleeding, infection and anosmia with neurological complications being extremely rare [1]. However, nasal trauma following a successful septorhinoplasty may lead to persistent nasal deformity resulting in a failed procedure. Therefore, one of the most important postoperative instructions given to the patient is to avoid nasal trauma.

Multiple sclerosis (MS) is the most common cause of neurological disability in young adults, with an estimated incidence of <5/100,000 [2]. Although it can develop at any age, it is most commonly diagnosed in people in the second and third decades of life and is about two to three times more common in women than in men. Furthermore, MS is one of the most common causes of disability in younger adults [2].

We present an unusual case of a young woman, who after a successful septorhinoplasty, experienced blunt nasal trauma associated with neurological symptoms and was subsequently diagnosed with MS.

## Case presentation

A 24-year-old healthy woman presented with difficulty breathing and dissatisfaction with her facial appearance. She had a history of childhood trauma resulting in nasal septum deviation and external nasal deformity. Four months after a successful and uneventful septorhinoplasty, she presented to the emergency department with blunt nasal trauma resulting in a septal hematoma, which was drained successfully; the patient was discharged with no adverse sequelae.

Four months later, the patient sustained nasal trauma again, this time accompanied by clear nasal discharge, raising suspicion of cerebrospinal fluid (CSF) leak. The patient was discharged after managing the nasal injury, as the CT brain showed an intact cribriform plate with no evidence of a CSF leak. Ten days later, she presented at the emergency department with dizziness and an unstable gait. She also had complaints of paresthesia for the past two months, beginning in her right hand and progressing to the right shoulder, arm and leg, associated with some difficulty in the execution of movements in the first and second finger of the right hand. Her right leg was quite stiff with difficulty in walking. On close inquiry, she gave history of pain in the right eye and double vision many months back, which had resolved spontaneously. Examination showed a positive Romberg’s and Lhermitte’s sign, with right-sided sensory impairment.

**Figure 1:**
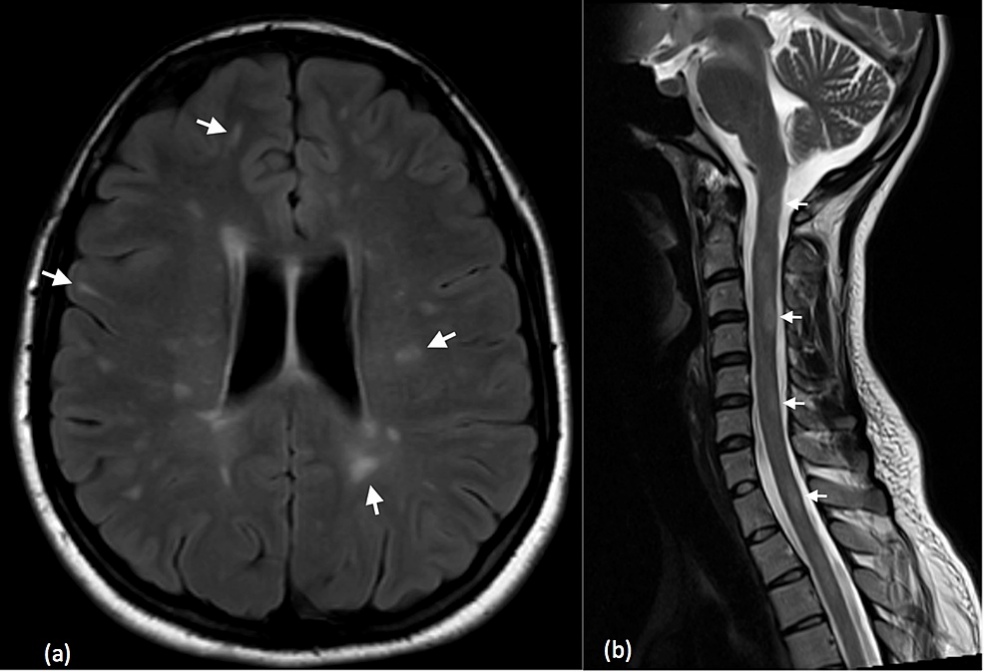
(a) Axial FLAIR image of the brain demonstrating multiple bilateral periventricular, deep white matter and subcortical hyperintense lesions (small white arrows), (b) sagittal T2-weighted images of cervical spine demonstrating multiple hyperintense intramedullary lesions (small white arrows) at different levels. FLAIR: fluid-attenuated inversion recovery.

Magnetic resonance imaging (MRI) of the brain, cervical and thoracic spine demonstrated demyelinating lesions in the brain and cervical segment of the spinal cord (Figure 1). Some of the lesions demonstrated enhancement on post gadolinium administration sequences, suggestive of active demyelinating diseases like MS. A lumbar puncture was performed which demonstrated the presence of oligoclonal bands in the CSF. The diagnosis of MS was confirmed by a neurologist and treatment was initiated. 

The initial neurological symptoms have largely vanished with only persistent light paresthesia in the right hand. Two years later she has had no new symptoms and continues with the same medication with good tolerance.

## Discussion

Nasal obstruction is associated with deviation of the septum, hypertrophy of inferior turbinate and ineffectiveness of external and internal nasal valves. Septorhinoplasty is one of the most common otorhinolaryngological procedures aimed at relieving the nasal obstruction effectively. Comprehensive preoperative evaluation of the patient, particularly nasal anatomy is very important. An accurate preoperative plan is devised only after accurately identifying the nasal abnormality and discussing the expected results with the patient. However, good postoperative care is even more crucial in achieving a successful surgical outcome. One of the most important instructions given to the patient is to be gentle in the nasal area and avoid any injury or manipulation. Patients are expected to be very cautious after septorhinoplasty as even simple nasal injuries at this critical juncture may result in procedure failure with a lifelong nasal deformity. While septorhinoplasty may be associated with complications like bleeding, infection, and anosmia, serious neurological sequelae such as traumatic CSF rhinorrhea are quite rare [3].

MS is an inflammatory demyelinating disease of the central nervous system with a wide array of neurological symptoms associated with progressive disability [1]. It is a multifactorial disorder attributed to complex interactions between susceptibility genes and environmental factors including low serum levels of vitamin D, smoking, childhood obesity, and infection with the Epstein-Barr virus and possibly trauma [1]. Breakdown of the blood-brain barrier, exposing neuronal tissue to inflammatory cytokines and neurotransmitters is postulated as a likely mechanism for the development of MS [4]. One of the hypotheses is that physical trauma, particularly involving the spinal cord and/or the brain, may cause a disruption of the blood-brain barrier, which in turn could lead to the development of MS plaques in those who are genetically at risk of developing the disease [5]. However, the high frequency of blood-brain barrier disruption in MS has also been observed in patients without preceding trauma, or conversely many individuals do not develop MS despite a history of traumatic injury [6]. Indeed, a recent meta-analysis has shown no significant association between physical trauma and MS [7]. 

In the case of our patient, she presented with two episodes of blunt nasal trauma after the surgery; the second one was accompanied by suspected CSF rhinorrhea and neurological symptoms, raising the suspicion of skull base injury. However, subsequent investigations established a definitive diagnosis of MS with no evidence of a septorhinoplasty-related neurological complication. It is most likely that the repeated nasal trauma in the post-surgical period resulted from the unsteady gait in this patient due to underlying but undiagnosed MS. While MS can occur at any age but is commonest in young people, particularly women, and can present with a wide array of symptoms including optic neuritis, diplopia, vertigo, sensory loss, motor weakness, gait disturbance and limb ataxia, in most cases with a remitting relapsing pattern [2]. Most of these symptoms also overlap with the clinical presentation of neurological complications post septorhinoplasty. Unless there is a high index of suspicion for an underlying neurological condition in a patient presenting with such symptoms post-operatively, an uncommon but disabling disorder such as MS may be missed.

## Conclusions

In conclusion, it is important to have an open mind when it comes to the differential diagnosis in unusual events postoperatively. When a patient presents with multiple episodes of nasal trauma post septorhinoplasty, an underlying neurological disorder should be strongly suspected and investigated, especially in young patients.

## References

[1] Dąbrowska-Bień J, Skarżyński PH, Gwizdalska I, Łazęcka K, Skarżyński H (2018). Complications in septoplasty based on a large group of 5639 patients. Eur Arch Otorhinolaryngol.

[2] Dobson R, Giovannoni G (2019). Multiple sclerosis - a review. Eur J Neurol.

[3] Soni RS, Choudhry OJ, Liu JK, Eloy JA (2013). Postoperative cerebrospinal fluid leak after septoplasty: a potential complication of occult anterior skull base encephalocele. Allergy Rhinol.

[4] Sweeney MD, Sagare AP, Zlokovic BV (2018). Blood-brain barrier breakdown in Alzheimer disease and other neurodegenerative disorders. Nat Rev Neurol.

[5] Lunny CA, Fraser SN, Knopp-Sihota JA (2014). Physical trauma and risk of multiple sclerosis: a systematic review and meta-analysis of observational studies. J Neurol Sci.

[6] Siva A, Radhakrishnan K, Kurland LT, O'Brien PC, Swanson JW, Rodriguez M (1993). Trauma and multiple sclerosis: a population-based cohort study from Olmsted County, Minnesota. Neurology.

[7] Warren SA, Olivo SA, Contreras JF, Turpin KV, Gross DP, Carroll LJ, Warren KG (2013). Traumatic injury and multiple sclerosis: a systematic review and meta-analysis. Can J Neurol Sci.

